# Polymorphous low-grade neuroepithelial tumor of the young with FGFR3-TACC3 fusion mimicking high-grade glioma: case report and series of high-grade correlates

**DOI:** 10.3389/fonc.2023.1307591

**Published:** 2023-11-21

**Authors:** Danielle Golub, Daniel G. Lynch, Peter C. Pan, Benjamin Liechty, Cheyanne Slocum, Tejus Bale, David J. Pisapia, Rupa Juthani

**Affiliations:** ^1^ Department of Neurosurgery, Weill Cornell Medicine, New York, NY, United States; ^2^ Department of Neurosurgery, Northwell Health, Manhasset, NY, United States; ^3^ Zucker School of Medicine at Hofstra/Northwell Health, Hempstead, NY, United States; ^4^ Department of Neurology, Weill Cornell Medicine, New York, NY, United States; ^5^ Department of Neurology, Columbia University, New York, NY, United States; ^6^ Department of Pathology, Weill Cornell Medicine, New York, NY, United States; ^7^ Department of Pathology, Memorial Sloan Kettering Cancer Center, New York, NY, United States

**Keywords:** FGFR fusion, glioblastoma, glioma molecular drivers, high-grade glioma, PLNTY

## Abstract

**Background:**

Polymorphous low-grade neuroepithelial tumor of the young (PLNTY) is a recently described entity that can mimic high-grade glioma (HGG) in histologic and molecular features; however, factors predicting aggressive behavior in these tumors are unclear.

**Methods:**

We present an indolent neuroepithelial neoplasm in a 59-year-old female with imaging initially suggestive of HGG, and a series of adult patients with HGG harboring FGFR3-TACC3 fusions are also presented for comparison.

**Results:**

Pathology in the case patient revealed low-grade cytomorphology, microcalcifications, unusual neovascularization, and a low proliferation index. The lesion was diffusely CD34+ and harbored an FGFR3-TACC3 fusion and TERT promoter mutation. A diagnosis of PLNTY was therefore favored and the patient was observed with no progression at 15-month follow-up. In patients with HGG with FGFR3-TACC3 fusions, molecular findings included IDH-wildtype status, absence of 1p19q codeletion, CDKN2A loss, TERT promoter mutations and lack of MGMT promoter methylation. These patients demonstrated a median 15-month overall survival and a 6-month progression-free survival.

**Conclusion:**

PLNTY is a rare low-grade entity that can display characteristics of HGG, particularly in adults. Presence of FGFR3-TACC3 fusions and other high-grade features should raise concern for a more malignant precursor lesion when a diagnosis of PLNTY is considered.

## 1 Introduction

Low-grade epilepsy-associated neuroepithelial tumors (LEATs) are a diverse set of epileptogenic neurodevelopmental lesions along a broad histological glial—glioneuronal spectrum that has made them historically difficult to classify (1, 2). Polymorphous low-grade neuroepithelial tumor of the young (PLNTY) represents the most recently recognized LEAT entity in the latest WHO classification, defined by oligodendroglioma-like cellular features, an infiltrative growth pattern, diffuse CD34 immunoreactivity, and frequent MAPK pathway aberrations—in particular, BRAF V600E and FGFR isoform fusion alterations (3, 4). On MRI, PLNTY can be difficult to distinguish from oligodendroglioma, DNET, or focal cortical dysplasia; it typically presents as a focal FLAIR hyperintensity with rare nodular enhancement and/or cystic components (5, 6). On CT, however, distinctive macrocalcifications are frequently observed (6). Increasing reports of LEATs consistent with PLNTY have helped to refine these defining characteristics, but the majority of reports to date have presented pediatric and young adult patients. Rare reports of PLNTY in middle-aged patients are partially explained by the lesions’ localization in the non-dominant hemisphere, likely prolonging the asymptomatic period (7, 8). Additionally, the differential diagnosis of heterogeneously enhancing cortical lesions in older patients generally prioritizes higher-grade gliomas over low-grade congenital lesions, further complicating the correct diagnosis of PLNTY in adults.

The FGFR3-TACC3 fusion, seen in a variety of solid tumors, produces a constitutively active fibroblast growth factor 3 receptor that leads to the upregulation of RAS-MAPK, PI3K-AKT, and JAK/STAT pathways responsible for increased cellular proliferation, migration, and angiogenesis (9). In high-grade glioma (HGG), this fusion alteration has been associated with amplification of cell cycle-related genes and decreased survival (10), however the fusion’s impact on survival invasiveness of low-grade pathologies such as PLNTY is not well-understood. Furthermore, it is unclear if the presence of the FGFR3-TACC3 fusion in an adult patient with PLNTY confers increased malignant potential, warranting adjuvant therapy. The concomitant presence of molecular findings common to HGGs with histologic findings and a clinical course consistent with a low-grade pathology such as PLNTY is unique and highlights some of the potential limitations of current molecular diagnostic criteria.

In this series, we describe an unusual case of adult PLNTY with an FGFR3-TACC3 fusion alteration that harbored histological and molecular features associated with HGG, but displayed a benign clinical course. We compare findings in this case to a review of published PLNTY cases to identify common and unique histologic and molecular features. We further contrast the molecular similarities and differences in this case of PLNTY to a corresponding series of 8 gliomas harboring FGFR3-TACC3 fusions identified by targeted sequencing panels. Finally, we assess the clinical outcomes including progression-free and overall survival in patients with glioblastoma (GBM) harboring the FGFR3-TACC3 fusion. This series aims to highlight a unique case of PLNTY and to identify potential limitations in our molecular diagnostic criteria for GBM.

## 2 Case presentation: PLNTY with high-grade features

**Figure 1:**
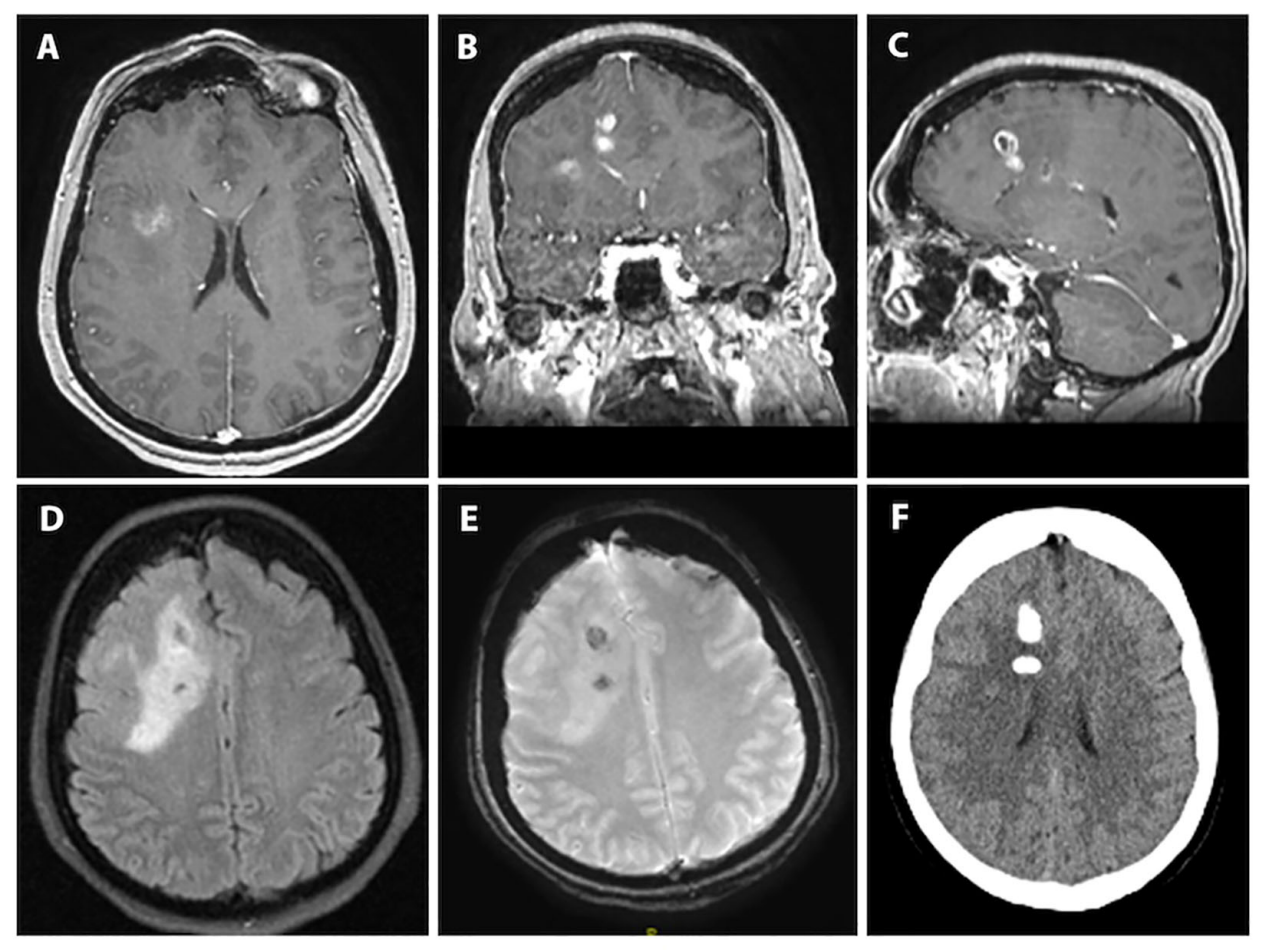
Preoperative imaging for index case of PLNTY with high-grade features. **(A-C)**: T1-weighted axial, coronal and sagittal sequences with contrast demonstrating multifocal enhancement involving the right frontal lobe and cingulate gyrus. **(D)**: T2/FLAIR-weighted axial sequence showing the bulk of the lesion to be T2/FLAIR hyperintense with clear mass effect and some surrounding vasogenic edema. **(E)**: Susceptibility-weighted imaging showing two areas susceptibility likely corresponding to intralesional calcification given the matching hyperdensities seen on **(F)**: axial computed-tomography imaging.

A 59-year-old, right-handed female presented with new onset generalized tonic-clonic seizures. CT demonstrated multiple masses associated with calcification in the right frontal lobe with vasogenic edema (**Figure 1F**). Subsequent magnetic resonance imaging (MRI) revealed multifocal enhancing lesions with associated areas of susceptibility involving the right frontal lobe, cingulate, and underlying white matter, with partial calcification containing intrinsic T1 hyperintensity. There was infiltrative appearing T2 signal hyperintensity extending across the midline along the corpus callosum with mild associated mass effect (**Figures 1A-E**). No T2-FLAIR mismatch was apparent. Radiological findings were reviewed at a multi-disciplinary tumor board, and favored to represent a glial neoplasm, either oligodendroglioma or high-grade astrocytoma. The patient underwent surgical resection, with post-operative MRI demonstrating a gross total resection of enhancing disease.

**Figure 2:**
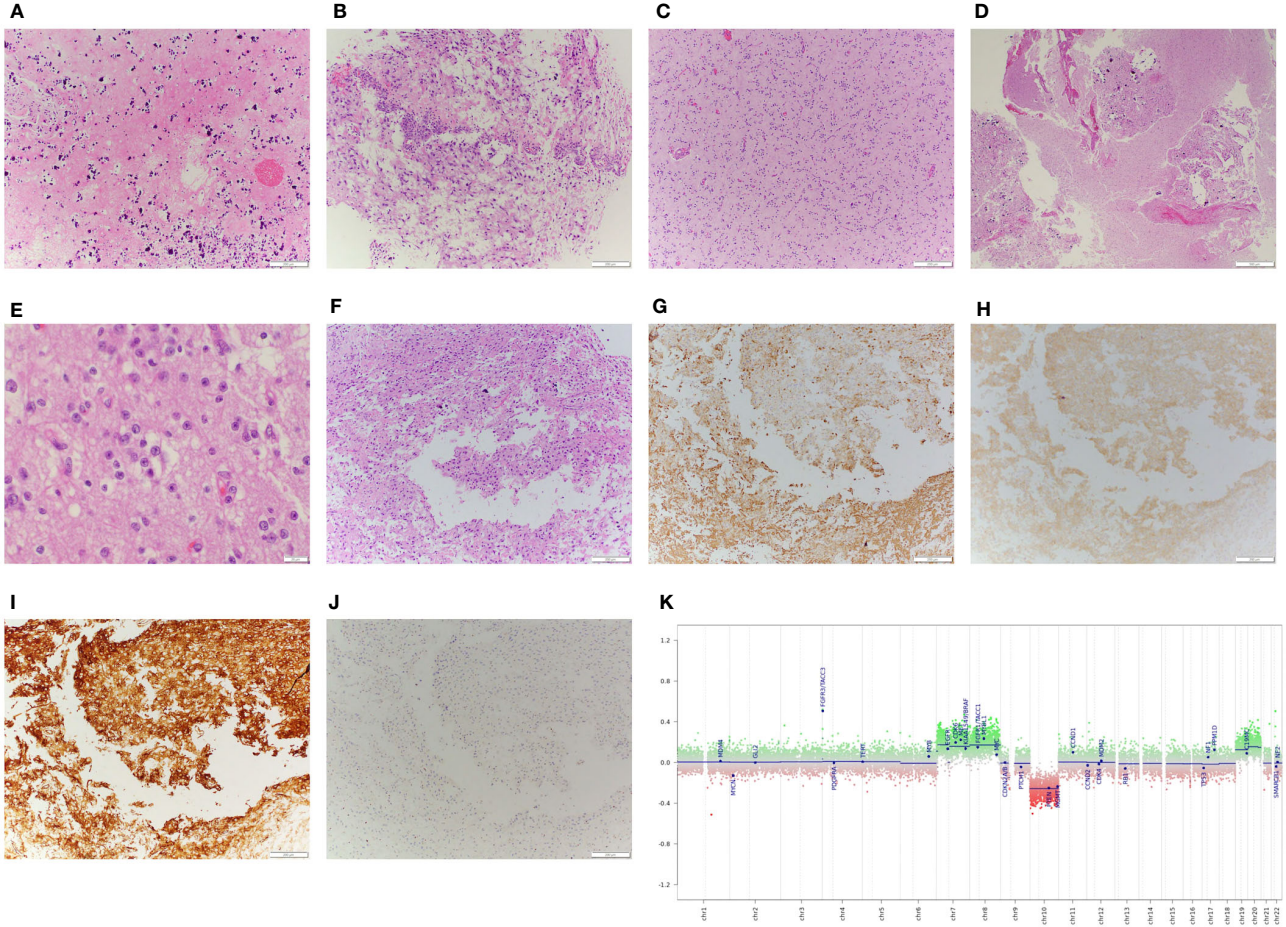
Pathological examination of surgical specimen including DNA methylation analysis. **(A)**: Histologic examination with geographic regions of necrosis with **(B)**: adjacent regions of sinusoidal neovascularization. The majority of tumor tissue demonstrated a cytologically bland neoplasm, **(C)**: comprised of regions with an infiltrative and **(D)**: occasionally more demarcated growth pattern, often associated with microcalcification. **(E)**: High power images of the tumor demonstrate some features suggestive of neurocytic differentiation, including open chromatin and prominent nucleoli. **(F)**: The greatest degree of nuclear atypia was seen in regions adjacent to the necrosis, which demonstrated variable immunoreactivity for **(G)**: GFAP and **(H)**: synaptophysin, with **(I)**: strong, diffuse staining for CD34. **(J)**: Of note, the Ki-67 proliferative index was low, <1% in atypical cells. **(K)**: Copy number analysis obtained from methylation array profiling data demonstrates whole chromosome gains of chromosomes 7, 8, 19, and 20, and loss of chromosome 10, and copy number gain of the FGFR3-TACC3 locus, consistent with the underlying fusion identified.

Initial frozen section was concerning for high-grade infiltrating glioma due to the presence of glial features, hyperplastic appearing vasculature, and necrosis. Permanent histologic sections, however, showed a moderately cellular neuroepithelial neoplasm with abundant microcalcification, with perivascular, perineuronal, and subpial growth (**Figures 2C, D**). Regions of necrosis and neovascularization were thought to represent intra-tumoral infarction rather than true tumoral necrosis or microvascular proliferation (**Figures 2A, B**). The remainder of the tumor appeared cytologically bland with cytoplasmic clearing and branched capillaries resembling oligodendroglioma (**Figure 2E**). Immunohistochemistry demonstrated strong staining for GFAP in some portions of the tumor and synaptophysin reactivity in other portions (**Figures 2F-H**). Notably, the tumor was diffusely, strongly positive for CD34 (**Figure 2I**). Tumor cells were non-reactive for both IDH1^R132H^ and p53, and showed preservation of ATRX. The Ki-67 proliferation index was <1% (**Figure 2J**). Of note, TERT promoter mutation and polysomy 7 without EGFR amplification was identified, and the MGMT promoter was unmethylated. Due to the unusual low-grade nature of this tumor, methylation array profiling was performed using the DKFZ brain tumor classifier (11). No match was obtained, and copy number analysis revealed polysomy of chromosomes 7 and 8 with monosomy of chromosome 10 (**Figure 2K**). Subsequent fusion analysis identified FGFR3-TACC3 fusion. Based on the low-grade morphology with oligodendroglioma-like components, CD34 immunopositivity, IDH-wildtype status, and absence of a match by methylation array profiling, an interpretation of a low-grade tumor such as PLNTY was favored (12).

The patient was discharged home on postoperative day 3 with no neurological deficits. Other than a single seizure episode two months postoperatively that resolved with the addition of a second anti-epileptic medication, she did well clinically with no recurrent symptomatology. She has been followed closely with MRIs every 3 months without evidence of recurrent or progressive disease with most recent follow-up at 15 months postoperatively.

## 3 Case series: FGFR3-TACC3 fusions in HGG

### 3.1 Methods

All HGGs treated at Weill Cornell Medicine found to have FGFR3-TACC3 fusion by targeted sequencing (Oncomine Comprehensive Panel v2, FoundationOne) were included in this study under an IRB-approved protocol. Tumor characteristics (including radiographic features, location, histopathology, and molecular analysis), treatment characteristics (including extent-of-resection, chemotherapy, and radiation therapy), and clinical outcomes (including progression-free survival and overall survival), were retrospectively reviewed and evaluated.

### 3.2 Results

A total of eight cases of GBM with FGFR3-TACC3 fusion were identified including two male and six female patients. Median age at diagnosis was 64 years (range 41-74). Seven of these tumors met histopathologic criteria for GBM on initial tissue analysis. All were IDH-wildtype, and 7/8 (87.5%) were MGMT unmethylated, while 1/8 (12.5%) harbored MGMT promoter methylation. Five tumors were located in the frontal lobe, 2 tumors in the parietal lobe, and 1 tumor in the temporal lobe. Gross total resection was achieved in 5 cases; the remainder were either sub-totally resected (1) or biopsied (2). All cases were treated with standard external beam radiotherapy and concurrent temozolomide following surgery. Half of the cases were treated with 59.4-60 Gy in 30-33 fractions, and the other half were treated with a hypofractionated course of 40.05 Gy in 15 fractions.

**Figure 3:**
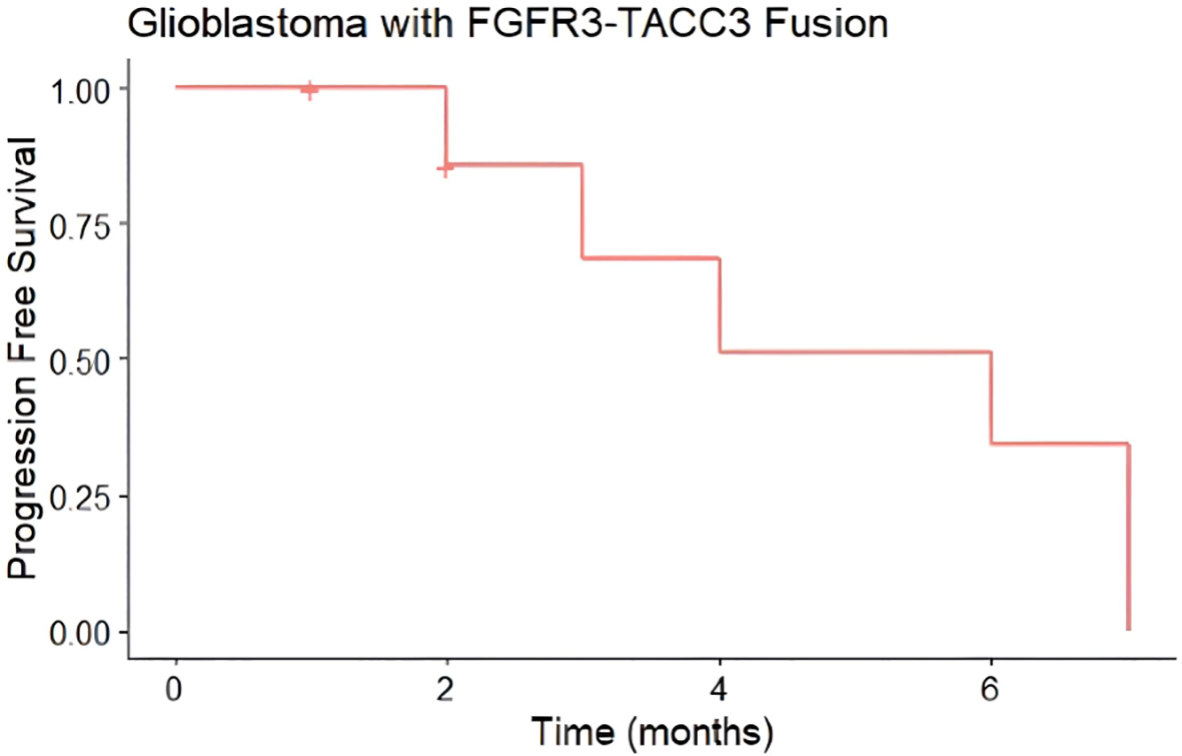
Progression-Free Survival (PFS) of glioblastomas from case series with FGFR3-TACC3 fusion. Median progression-free survival was 6 months (n = 8, 6 events). Hashmark indicates censored.

**Figure 4:**
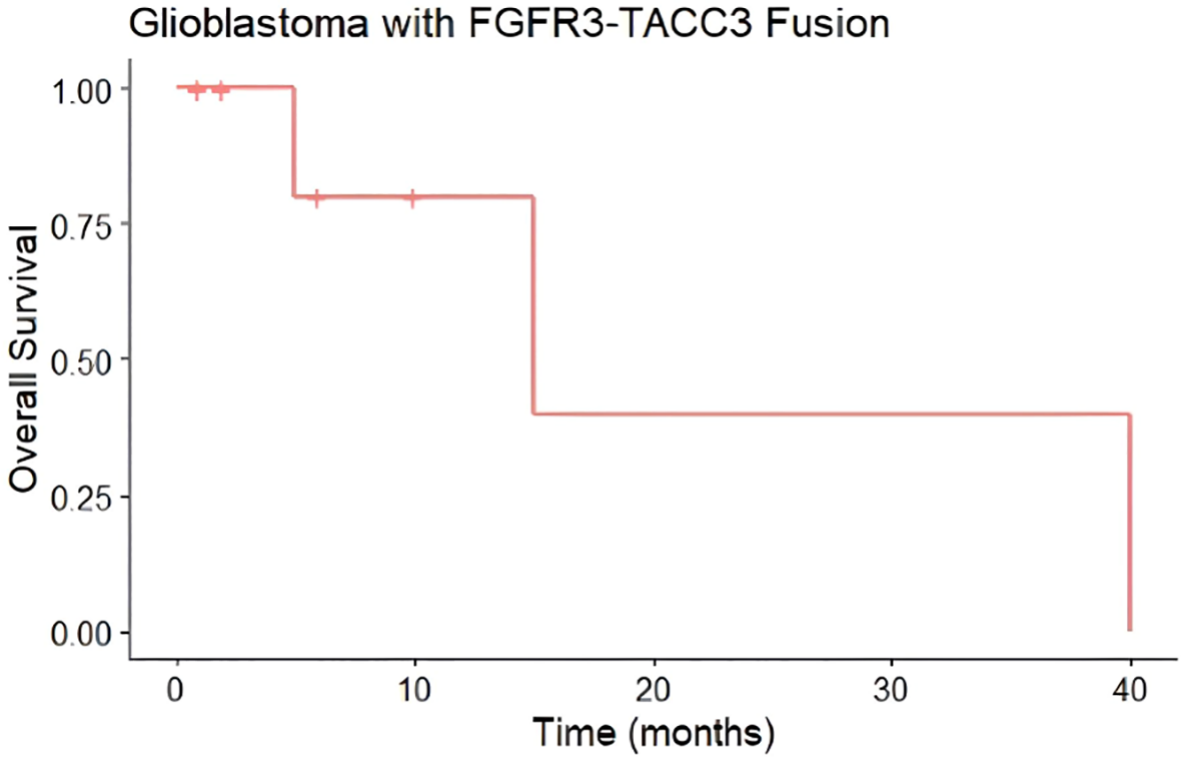
Overall Survival (OS) of glioblastomas from case series with FGFR3-TACC3 fusion. Median overall survival of 15 months (n=8, 3 events). Hashmark indicates censored.

Median progression-free survival in this cohort was 6 months (**Figure 3**), while median overall survival was 15 months (**Figure 4**). Three patients progressed rapidly after initial chemoradiation and were not treated further with adjuvant temozolomide chemotherapy: A 63-year-old man with a left frontal GBM who underwent subtotal resection followed by temozolomide and a radiation dose of 59.4 Gy in 33 fractions, who elected hospice care at 3 months postoperatively and ultimately died 15 months postoperatively, and two other patients who were lost to follow-up after transitioning to hospice; a 71-year-old woman with a gross totally-resected right frontal GBM and a 61-year-old woman with a biopsied left parietal GBM. Both received temozolomide with radiation dose of 40.05 Gy in 15 fractions and transitioned to hospice before adjuvant chemotherapy.

## 4 Discussion

### 4.1 Histological and molecular characteristics of PLNTY

The term “polymorphous” was originally ascribed to PLNTYs because of their significant histological heterogeneity; while consistently demonstrating areas of oligodendroglioma-like cells with round nuclei and perinuclear halos, some PLNTY samples also have areas of vague perivascular ependymoma-like pseudorosetting and patchy regions of fibrillary, spindled astrocytic components (4). A consistent finding across PLNTY lesions is strong, diffuse CD34 immunoreactivity (4). CD34, a well-known intercellular adhesion protein and marker of hematopoietic stem cells, is also transiently expressed in early neural development (13). In addition to PLNTY, ganglioglioma, DNET, and pleomorphic xanthroastrocytoma (PXA) are often focally CD34+, albeit in a more heterogeneous distribution (14). The expression of CD34 across multiple pediatric lesions, particularly LEATs, suggests that these lesions may involve developmentally arrested or dysregulated neural progenitors. In fact, most of these CD34+ lesions, including PLNTY, are also frequently associated with regional cortical dysplasia (15). Accordingly, one of the largest series examining LEATs found that CD34 expression was associated with a significantly longer duration of epilepsy—further suggesting a congenital or developmental structural cause (16).

PLNTY also exhibits a collectively distinct genetic and epigenetic signature. Huse et al.’s original series identified frequent mutually exclusive genetic aberrations involving the MAP kinase pathway including BRAF V600E and FGFR2 or FGFR3 fusion transcripts (4). Since this seminal work, mutually exclusive BRAF and FGFR2 or FGFR3 mutations have been identified in nearly all reported cases of PLNTY (**Table 1**) (4, 6–8, 17–23). BRAF V600E is a constitutively active downstream effector in the MAPK pathway that is a widely implicated oncogenic driver in several cancers and an established pharmacogenetic target. Similarly, the FGFR2 and FGFR3 fusion transcripts constitutively dimerize to activate the MAPK pathway and are also potential therapeutic targets (24). While MAPK pathway alterations clearly play a critical driver role in PLNTY, the same can be said of a majority of LEATs (5) and of several other types of cancers. However, genome-wide methylation profiling has established that PLNTY exhibits a distinct DNA methylation signature most closely related to that of ganglioglioma; in fact, when applied to a wider set of previously profiled tumors, Huse et al. found that two additional lesions initially diagnosed as ganglioglioma and low-grade glioma, NOS actually clustered best with the PLNTY methylation signature, and furthermore harbored consistent FGFR2 fusion alterations (4). While most LEAT subtypes, including PLNTY, rely on MAPK pathway activation, distinct DNA methylation signatures and histological characteristics suggest etiological differences based on the differentiation state of the lesional cell of origin.

**Table 1:** Review of Cases of PLNTY in the Literature.

Studies	No. of Cases	Age	Gender	Onset of Epilepsy	Lesion Localization	Histological Features	OLIG2+	GFAP+	CD34+	Ki67	BRAF Mutation	FGFR Fusion Transcript
**Huse et al.** (2017) (4)	10	4-32	4M, 6F	3-22	6 R temporal 1 L temporal 2 R occipital 1 R frontal	* All: Oligodendroglioma-like cells with compact nuclei and perinuclear halos * 9: Patchy fibrillary and spindled astrocytic features * All: Patchy perivascular pseudorosettes * 9: Calcifications	All: Strong	All: Patchy	All: Strong, diffuse; +regional cortex	8: <1% 1: 3% 1: 5%	3 BRAF V600E	1 FGFR3-TACC3 1 FGFR2-KIAA1598 1 FGFR2-CTNNA3
**Bitar et al.** (2018) (17)	1	31	M	10	R temporal	* Oligodendroglioma-like cells with compact nuclei and perinuclear halos * Patchy fibrillary and spindled astrocytic features * Rare eosinophilic granular bodies		Patchy	Strong, diffuse; +regional cortex	1-2%	BRAF V600E	
**Riva et al.** (2018) (8)	1	57	M	No seizures	R frontal	* Oligodendroglioma-like cells with compact nuclei and perinuclear halos * Patchy perivascular pseudorosettes	Yes	Yes	Strong, diffuse; +regional cortex	2-3%		FGFR3-TACC3
**Gupta et al.** (2019) (18)	1	30	M	22	R middle temporal gyrus	* Oligodendroglioma-like cells with compact nuclei and perinuclear halos		Moderate staining between tumor cells	Strong, diffuse; +regional cortex	<1%	BRAF V600E	
**Johnson et al.** (2019) (6)	9	5-34	2M, 7F		3 R temporal 3 L temporal 1 R parietal 1 L parietal 1 3^rd^ ventricle	* All: Oligodendroglioma-like cells with compact nuclei and perinuclear halos * 8: Calcifications	All: Yes	All: Patchy	Diffuse in most cases, focal or patchy in some cases		4 BRAF V600E 1 BRAF fusion	2 FGFR2-KIAA1598 1 FGFR2 rearrangement
**Lelotte et al.** (2019) (19)	1	33	F	31	R temporal	* Oligodendroglioma-like cells with compact nuclei and perinuclear halos * Astrocytic and pilocytic regions * Patchy perivascular pseudorosettes	Yes	Yes	Strong, diffuse; +regional cortex	<1%	BRAF V600E	
**Sumdani et al.** (2019) (20)	1	19	M	19	R parietal	* Oligodendroglioma-like cells with compact nuclei and perinuclear halos * Calcifications			Strong, diffuse	<1%	BRAF V600E	
**Surrey et al.** (2019) (21)	6	7-16	4M, 2F		3 temporal 1 parietal 1 temporo-parietal 1 temporo-occipital	* All: Oligodendroglioma-like cells with compact nuclei and perinuclear halos * All: variable astrocytic features * 2: Rare eosinophilic granular bodies * 4: Calcifications			All: Strong, diffuse		3 BRAF V600E	1 FGFR2-CTNNA3 2 FGFR2-INA
**Benson et al.** (2020) (7)	1	44	F		L temporal	* All: Oligodendroglioma-like cells with round-to-ovoid nuclei and perinuclear halos * Calcifications	Strong		Strong, diffuse	<1%	BRAF V600E	
**Chen et al.** (2020) (22)	3	14-16	2M, 1F	13-14	1 R temporal 1 L temporal 1 R frontal	* All: Oligodendroglioma-like cells * All: Patchy spindled astrocytic features * All: Calcifications	All: Yes		All: Strong, diffuse		1 BRAF V600E	1 FGFR3-TACC3
**Bale et al.** (2021) (23)	1	15	F	15	L temporal	* Oligodendroglioma-like cells * Patchy perivascular pseudorosettes * Calcifications			Strong, diffuse	<2%		FGFR3-TACC3

### 4.2 FGFR3-TACC3 fusions in GBM

The FGFR3-TACC3 fusion identified in our index PLNTY case can been seen in 3-8% of GBMs (25, 26). Like its manifestation in PLNTY, FGFR3-TACC3 fusion is mutually exclusive with the more common receptor tyrosine kinase mutations in GBM such as EGFR, PDGFR, or MET (27). In glioma and GBM, FGFR3-TACC3 is also associated with wild-type IDH status, homozygous deletion of CDKN2A, amplification of CDK4 and MDM2, and decreased survival—sometimes despite lower grade features such as low proliferation indices (10, 28, 29). These characteristics were consistent in our FGFR3-TACC3-positive GBM series in which all 8 lesions were IDH-wildtype and were predominantly MGMT promoter unmethylated. In our series, survival was comparable to the median survival reported by Stupp et al., without clearly conferring a poorer prognosis (30). However, this comparison is limited by the small sample size in this study; larger series are necessary to make meaningful conclusions regarding the implication of the FGFR3-TACC3 fusion on survival in GBM.

In the first description of the subset of GBMs harboring the FGFR3-TACC3 fusion, Singh et al. suggested that the fusion alteration also played a role in increasing chromosomal instability and aneuploidy (26). Soon after, Parker et al. identified a critical loss of a 3’-untranslated region of FGFR3 in the fusion construct that confers resistance to regulation by microRNAs, and demonstrated constitutive activity of the receptor itself that engages downstream MAPK and PI3K signaling (27). Shared histological features of GBMs harboring FGFR3-TACC3 include nuclear monomorphism (similar to what is classically observed in oligodendroglioma), frequent microcalcifications, perivascular pseudorosettes, and CD34 ramified labeling—features also seen in PLNTY (28). Such distinctive similarities between PLNTY and GBM with FGFR3-TACC3 fusions may confound diagnosis and associated prognostic implications; it is conceivable that PLNTY with FGFR3-TACC3 fusion, particularly in adult patients, may act more aggressively, making correct diagnosis and determination of subsequent treatment critical. Indeed, FGFR rearrangements in the setting of CDKN2A/B loss and ATRX loss have been associated with more aggressive behavior in classically lower grade tumors such as pilocytic astrocytoma (31).

The FGFR3-TACC3 fusion has also been studied as a targetable mutation in GBM. In addition to positive data from a handful of preclinical studies (10, 26, 27), at least two pan-FGFR tyrosine kinase inhibitors, Erdafitinib (JNJ-42756493) and Dovitinib (TKI258), have demonstrated safety and some limited but promising clinical efficacy in phase I studies (32, 33). Whether there is a role for these adjuvant treatments in PLNTY remains to be determined.

### 4.3 Unique characteristics in late presentation of PLNTY: implications for molecular diagnoses and clinical management

This case of PLNTY demonstrates multiple irregularities not previously reported in the literature, with a comprehensive review of previously published cases summarized in **Table 1**. PLNTY is rare in the adult population, making this a unique case of PLNTY in a 59-year-old patient. Histologically, the areas of intra-tumoral infarct and regions mimicking neovascularization in the setting of an infiltrative-appearing tumor may initially suggest the diagnosis of a HGG or GBM. Moreover, the tumor was found to harbor a TERT promoter mutation, which in the setting of IDH-wildtype diffuse astrocytoma is now thought to be sufficient for a diagnosis of GBM (3, 34). However, given the remarkably low Ki-67 proliferation index, abundant microcalcifications suggestive of a long-standing process, oligodendroglioma-like features, strong and diffuse CD34 positivity, and the presence of an FGFR3-TACC3 fusion, a diagnosis of PLNTY was ultimately chosen in light of the latest diagnostic criteria from the fifth edition of the WHO Classification of Tumors of the Central Nervous System (3, 35). Additionally, while there were no high confidence matches in DNA methylation array analysis using the latest Heidelberg classifier (v12.5), the tumor matched closest to the methylation class “Low-grade glial/glioneuronal/neuroepithelial tumors” (calibration score 0.46).

While PLNTY is still rare enough that the associated literature and experience are still insufficient to confidently guide clinical management, the available cases do support an indolent course compared to GBM. Clinically, the patient has shown no evidence of recurrence despite lack of adjuvant treatment over a 15-month period, which strongly supports the diagnosis of a lower grade entity. Interestingly, the only other report of PLNTY in a patient over 50 also identified an FGFR3-TACC3 fusion and found a Ki-67 index of 2-3% (8). These similarities with respect to subtle high-grade features raise the possibility of a unique entity of PLNTY in adults that may require a more individualized approach including increased surveillance. Nevertheless, the misclassification of a high-grade tumor can be devastating, and extensive multi-institutional, multi-disciplinary discussion including neuropathology, neuroradiology, neuro-oncology, neurosurgery, and radiation oncology was employed prior to the determination that the tumor in this index case would be followed without adjuvant therapy. The patient and family were counseled on the rarity of this entity, and the possibility of a recurrence necessitating further treatment.

The identification of malignant behavior in FGFR3-altered low-grade tumors is likely multifactorial and may involve identifying additional alterations in DNA damage signaling and telomere maintenance. Interestingly, in our comprehensive review of reported PLNTY cases (**Table 1**), there was no previous report of a concomitant TERT promoter mutation as observed in our index case. Analysis of the MSK-IMPACT glioma cohort revealed no significant difference survival between FGFR3-altered gliomas with or without the TERT mutation; however, a statistically insignificant trend towards reduced median survival was observed when concurrent alterations in CDKN2A were present (**Supplementary Figure 1**) (36–38). Furthermore, no significant differences in survival were noted in this cohort with mutations in genes associated with p16-RB1 signaling (**Supplementary Figure 2**), p14-p53 signaling (**Supplementary Figure 3**), or telomere maintenance (**Supplementary Figure 4**). These analyses are limited both by small sample size and the multiple alterations included for each gene considered.

There has been one previous report in a pediatric patient of malignant transformation of a lesion with an FGFR3-TACC3 fusion originally diagnosed as PLNTY based on histological findings (23). Interestingly, despite the recurrent lesion’s glioblastoma-like features, the patient responded well to proton-based radiotherapy and temozolomide and remained recurrence-free at 34 months. The potential aggressiveness of PLNTY with FGFR-TACC fusion alterations contrasts the existing literature on PLNTY with BRAF V600E mutations. Although more often seen in adult male patients, BRAF V600E positive PLNTY cases have not demonstrated an increased propensity for recurrence or aggressiveness (39, 40). Furthermore, our literature review (**Table 1**) generally supports a pathological correlation between BRAF V600E expression and a lower mitotic index (all <1%). Nonetheless, while stability at 15-month follow-up is reassuring, it remains possible that recurrence could refute the original interpretation of the case lesion as PLNTY. Given that this tumor is rarely seen in the adult population and not previously described collectively with FGFR3-TACC3 fusion alteration, TERT promoter mutation, and polysomy 7, the potential for a more aggressive clinical course remains and necessitates close follow-up and counseling.

## 5 Conclusions

PLNTY is a rare entity that clinically and histologically can mimic HGG. While more commonly seen in the pediatric population, it should be considered in the differential for adult primary tumors. Characteristic genetic alterations may predispose to malignant transformation, in contrast to pediatric cases. Lesions found to have FGFR3-TACC3 fusions with histopathologic features most consistent with HGG clinically may be associated with a worse prognosis when coupled with mutations such as TERT promoter, but further studies are needed to better define potential differences in overall and progression-free survival based on the presence of this fusion with and without concurrent molecular alterations. Close attention to follow-up in low-grade lesions, such as PLNTY, is recommended, especially in adult patients with high-grade molecular characteristics given the potential consequences of withholding adjuvant therapy in the event of a truly higher-grade entity. A larger series of such cases with long-term follow-up and attention to molecular alterations may help elucidate the role and timing of adjuvant treatment.

## Data availability statement

The original contributions presented in the study are included in the article/**Supplementary Material**. Further inquiries can be directed to the corresponding author.

## Ethics statement

The studies involving humans were approved by Weill Cornell Institutional Review Board. The studies were conducted in accordance with the local legislation and institutional requirements. The participants provided their written informed consent to participate in this study. Written informed consent was obtained from the individual(s) for the publication of any potentially identifiable images or data included in this article.

## Author contributions

DG: Conceptualization, Formal Analysis, Methodology, Supervision, Visualization, Writing – original draft, Writing – review & editing. DL: Data curation, Formal Analysis, Methodology, Writing – original draft, Writing – review & editing. PP: Formal Analysis, Investigation, Resources, Supervision, Writing – review & editing. BL: Data curation, Formal Analysis, Supervision, Visualization, Writing – review & editing. CS: Data curation, Investigation, Writing – original draft. TB: Conceptualization, Data curation, Formal Analysis, Resources, Writing – review & editing. DP: Conceptualization, Formal Analysis, Investigation, Methodology, Supervision, Writing – review & editing. RJ: Conceptualization, Data curation, Methodology, Project administration, Supervision, Writing – original draft, Writing – review & editing.
