# Supplementary material for: Polymorphous low-grade neuroepithelial tumor of the young with FGFR3-TACC3 fusion mimicking high-grade glioma: case report and series of high-grade correlates
Source: Front Oncol. 2023 Nov 21;13:1307591. doi: 10.3389/fonc.2023.1307591 (PMC10698862; doi:10.3389/fonc.2023.1307591)

Supplementary Figure 1 – TERT and CDKN2A alterations

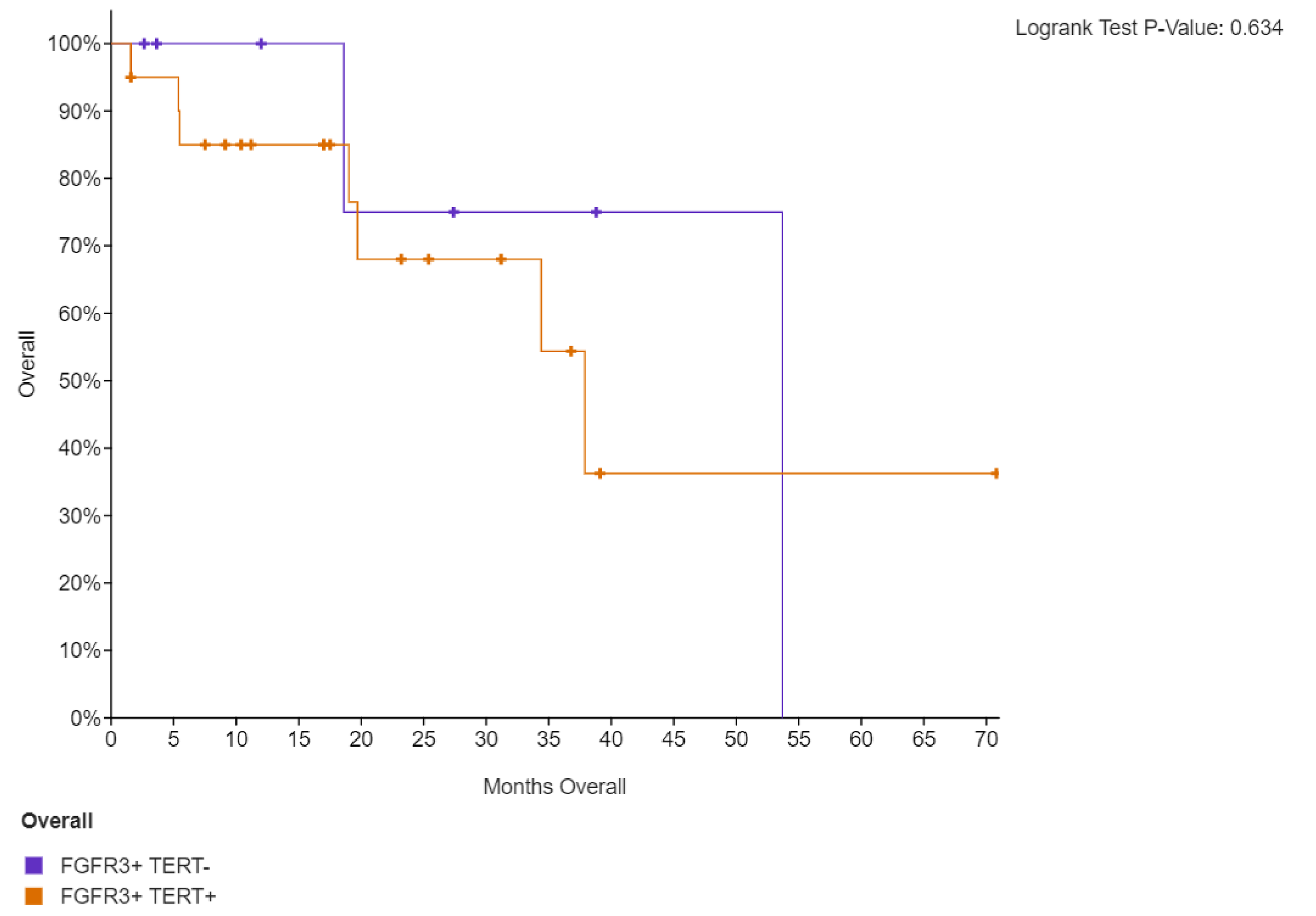

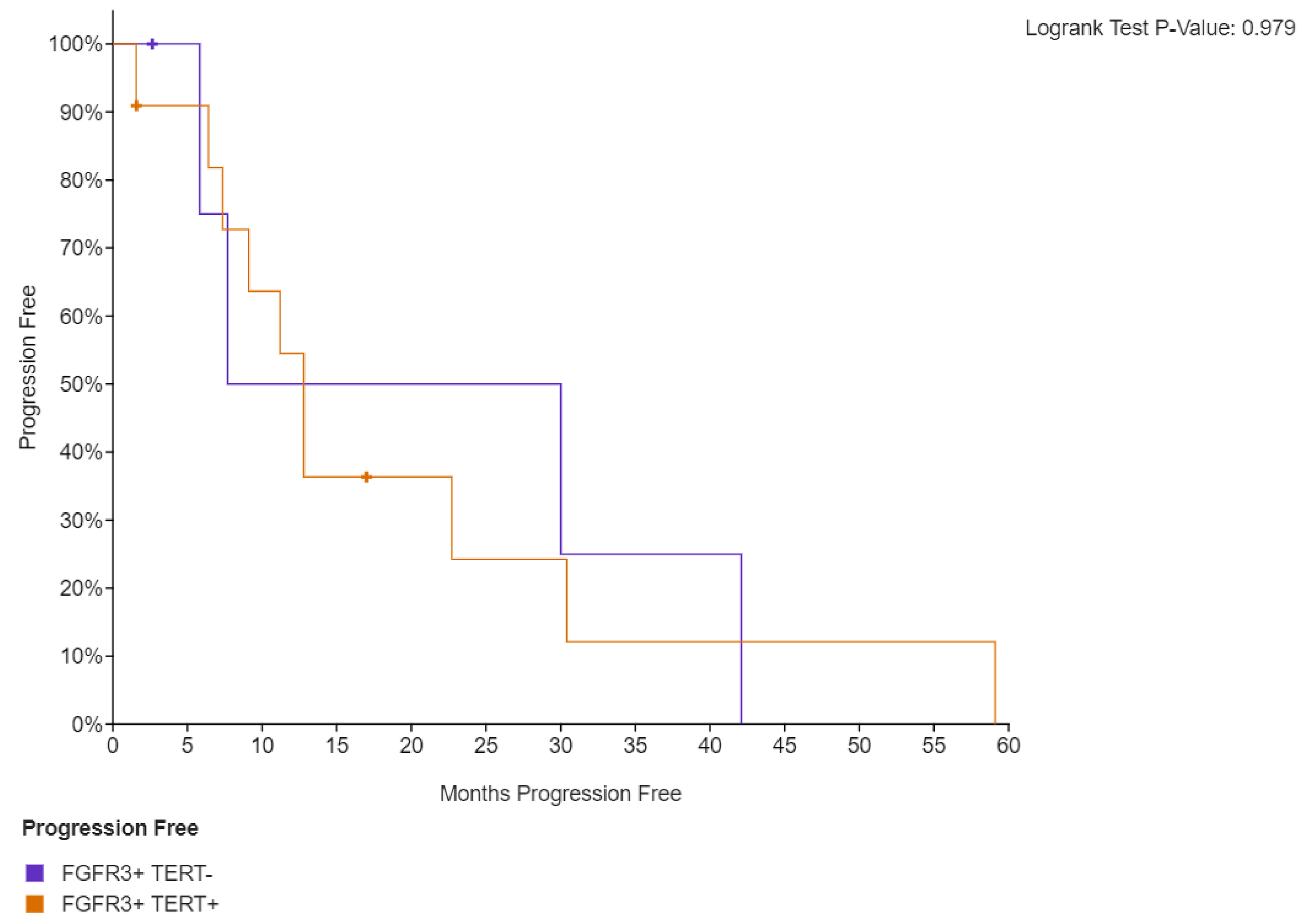

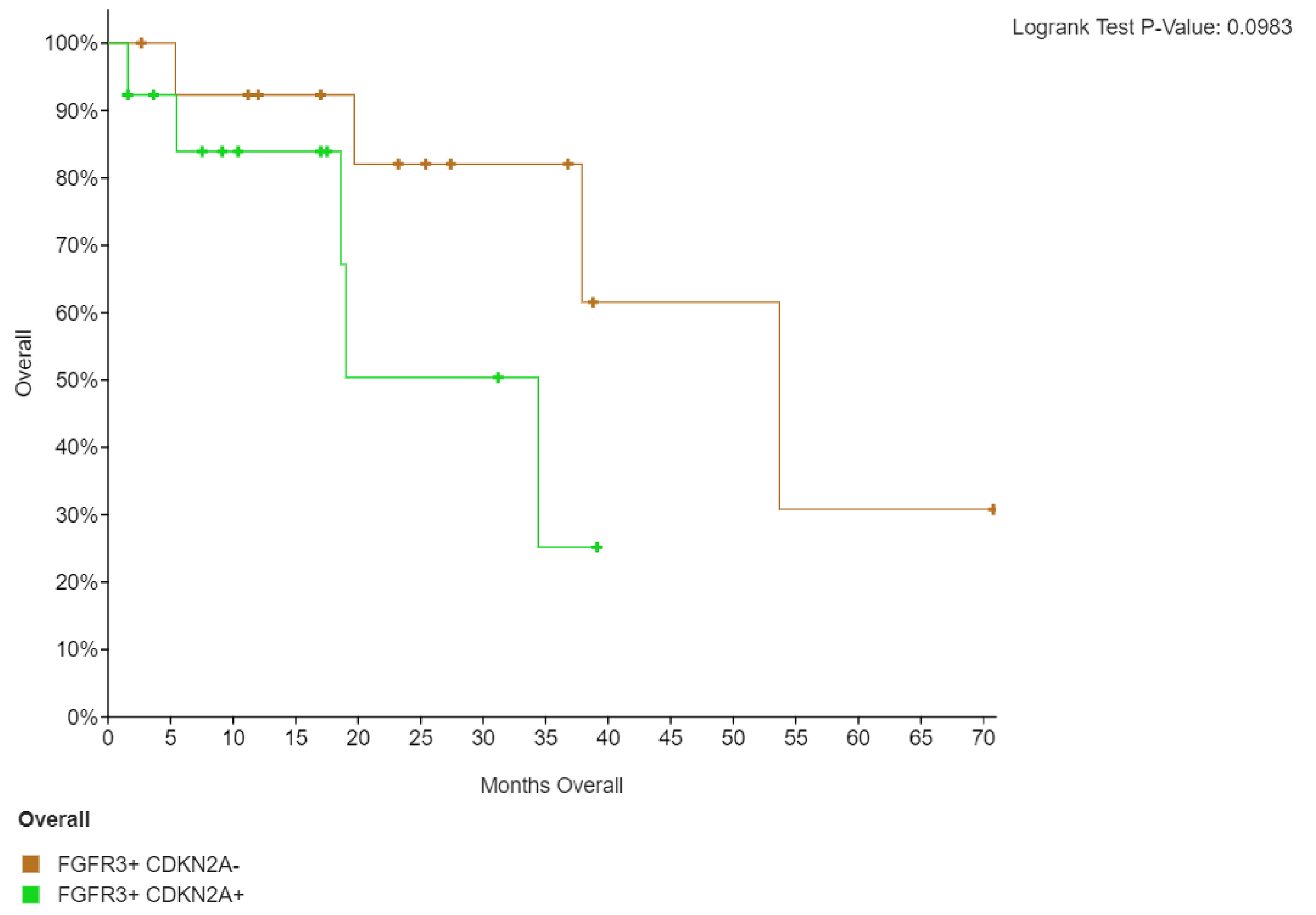

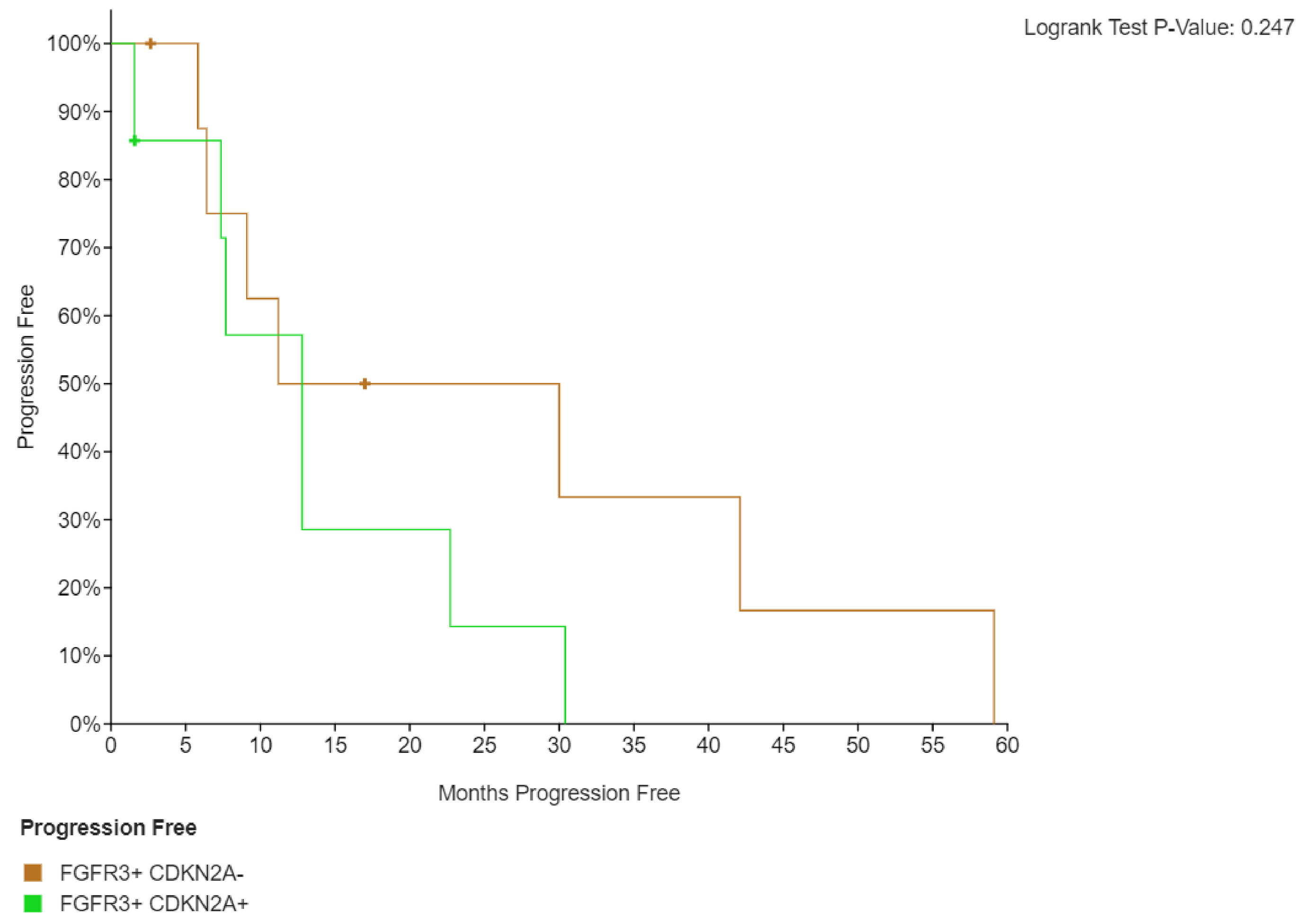

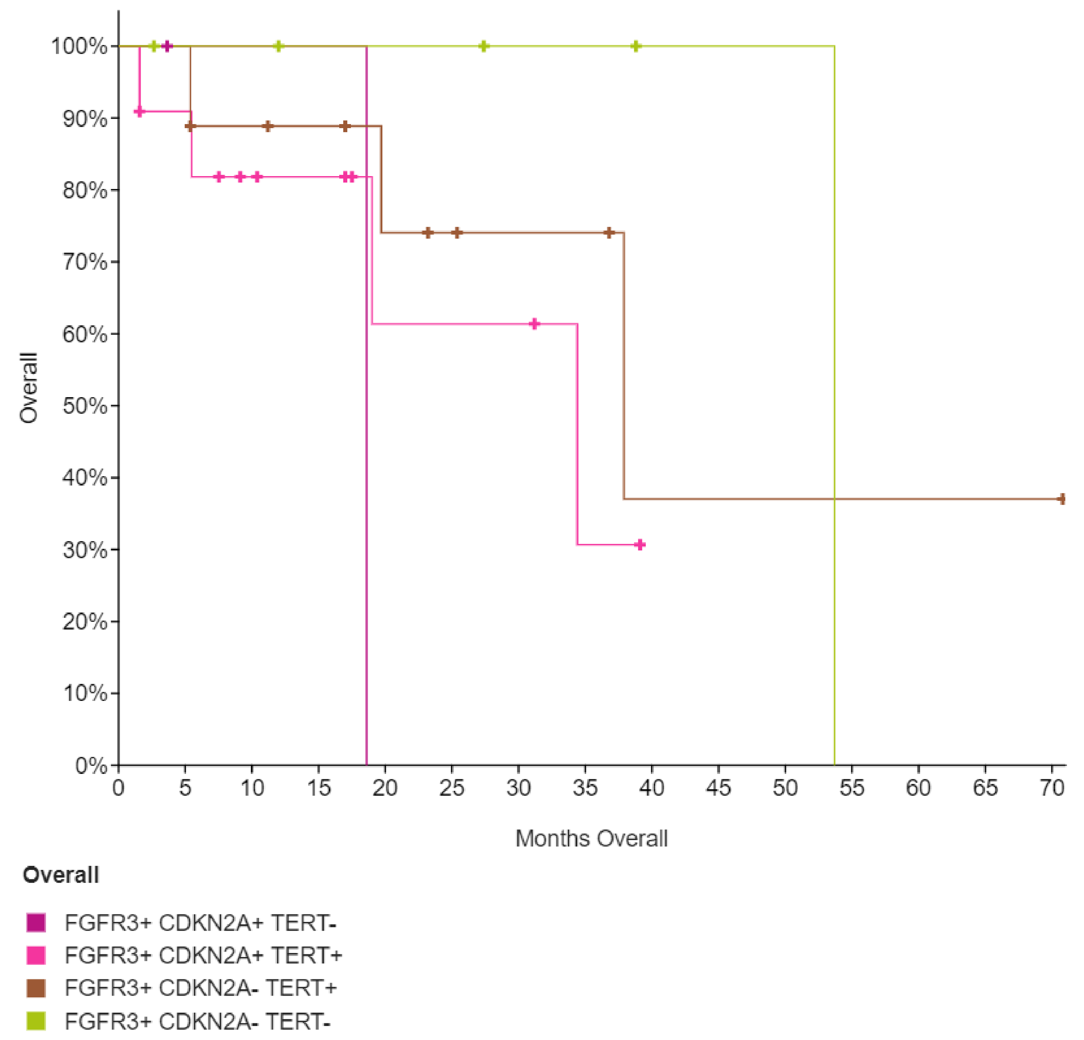

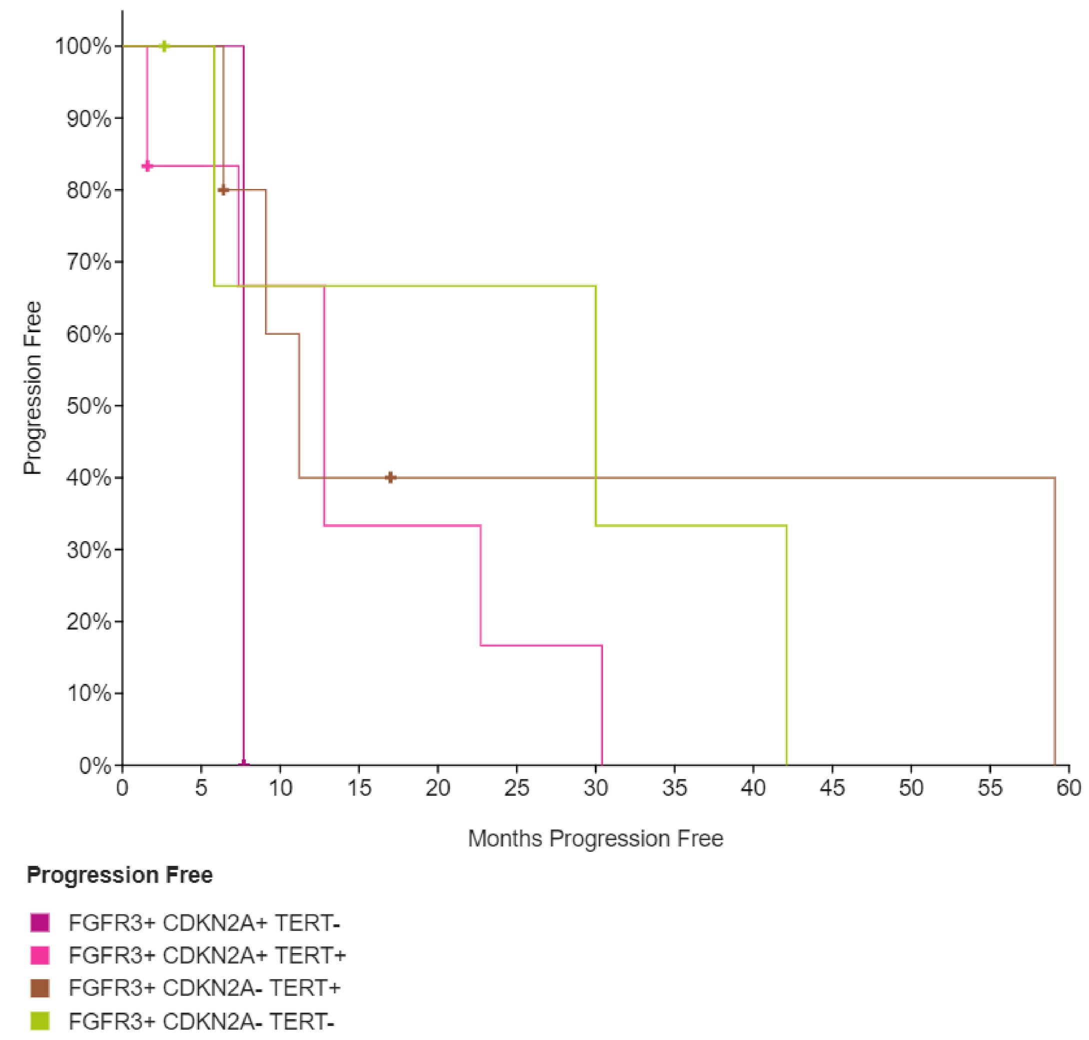

Supplementary Figure 2 – Other p16-RB1 pathway alterations

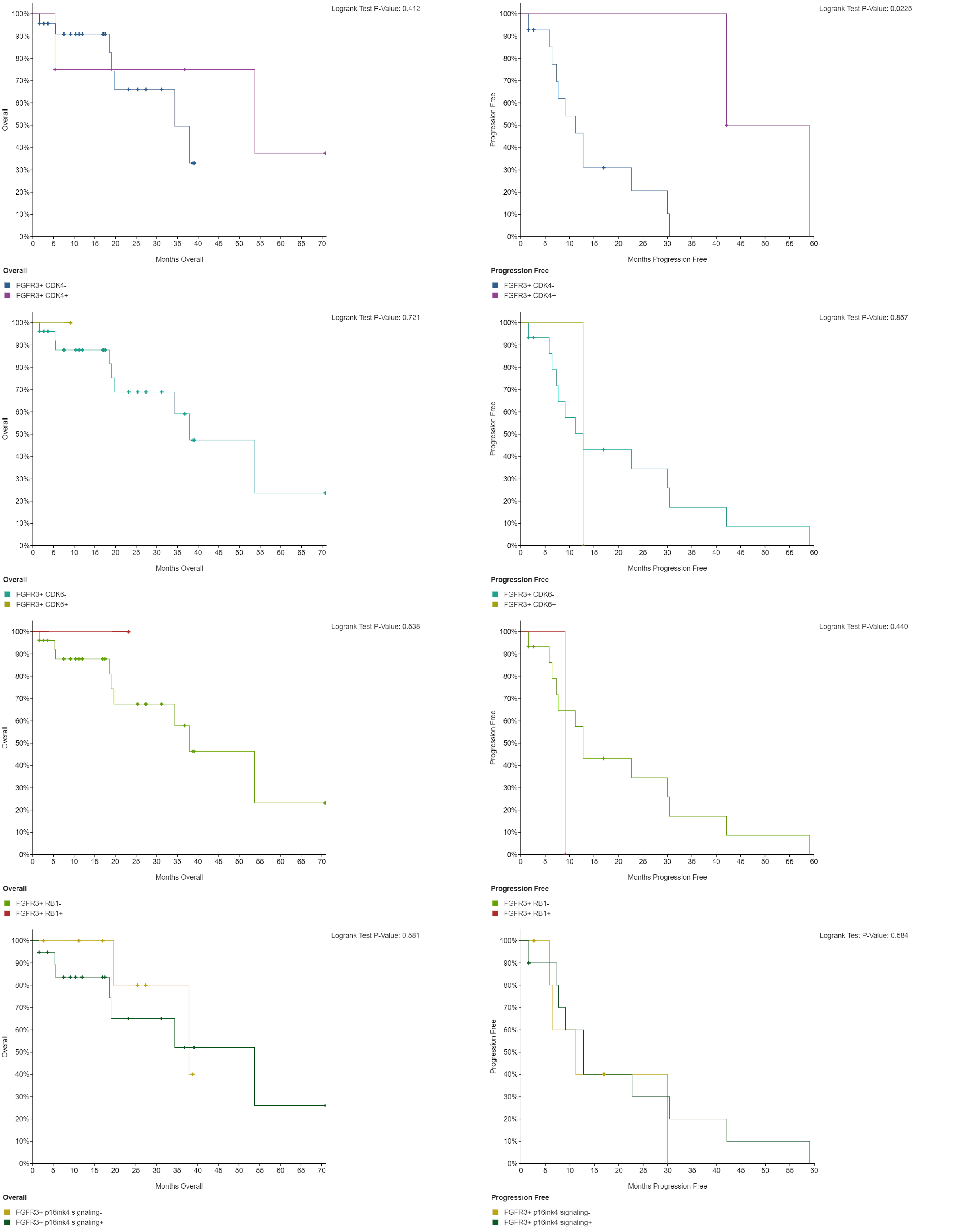

Supplementary Figure 3 – Other p14-p53 pathway alterations

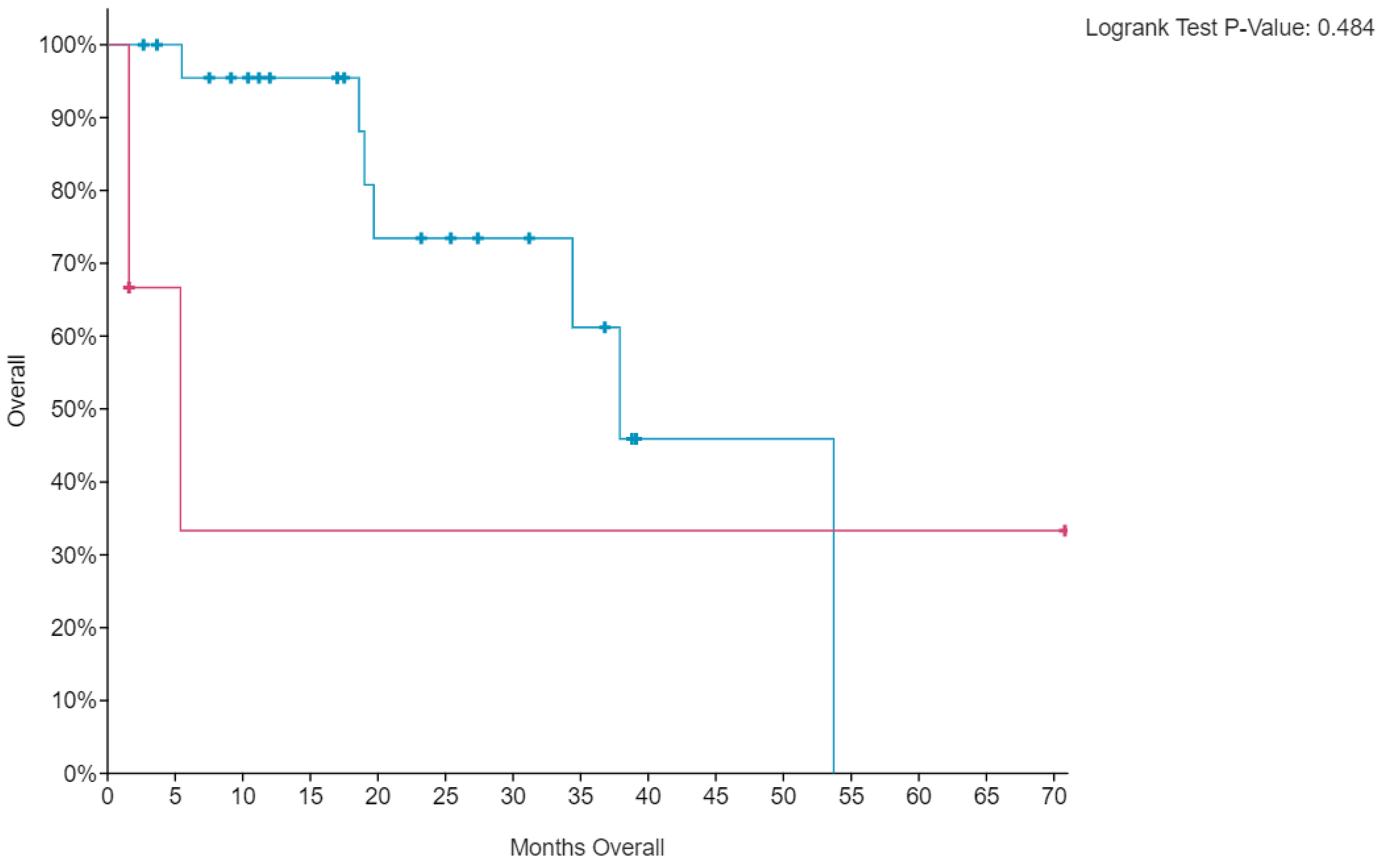

Overall

- FGFR3+ MDM2-
- FGFR3+ MDM2+

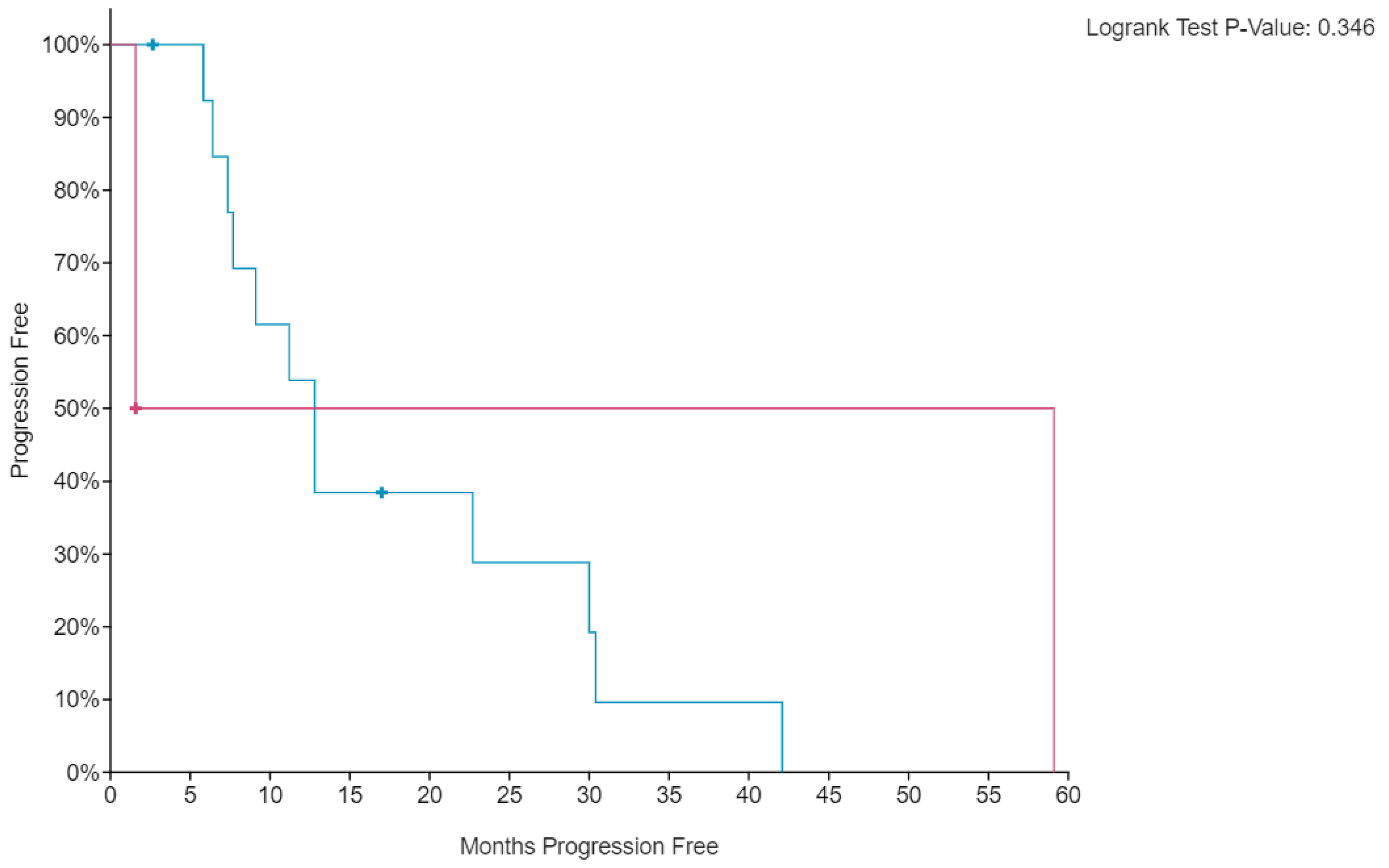

Progression Free

- FGFR3+ MDM2-
- FGFR3+ MDM2+

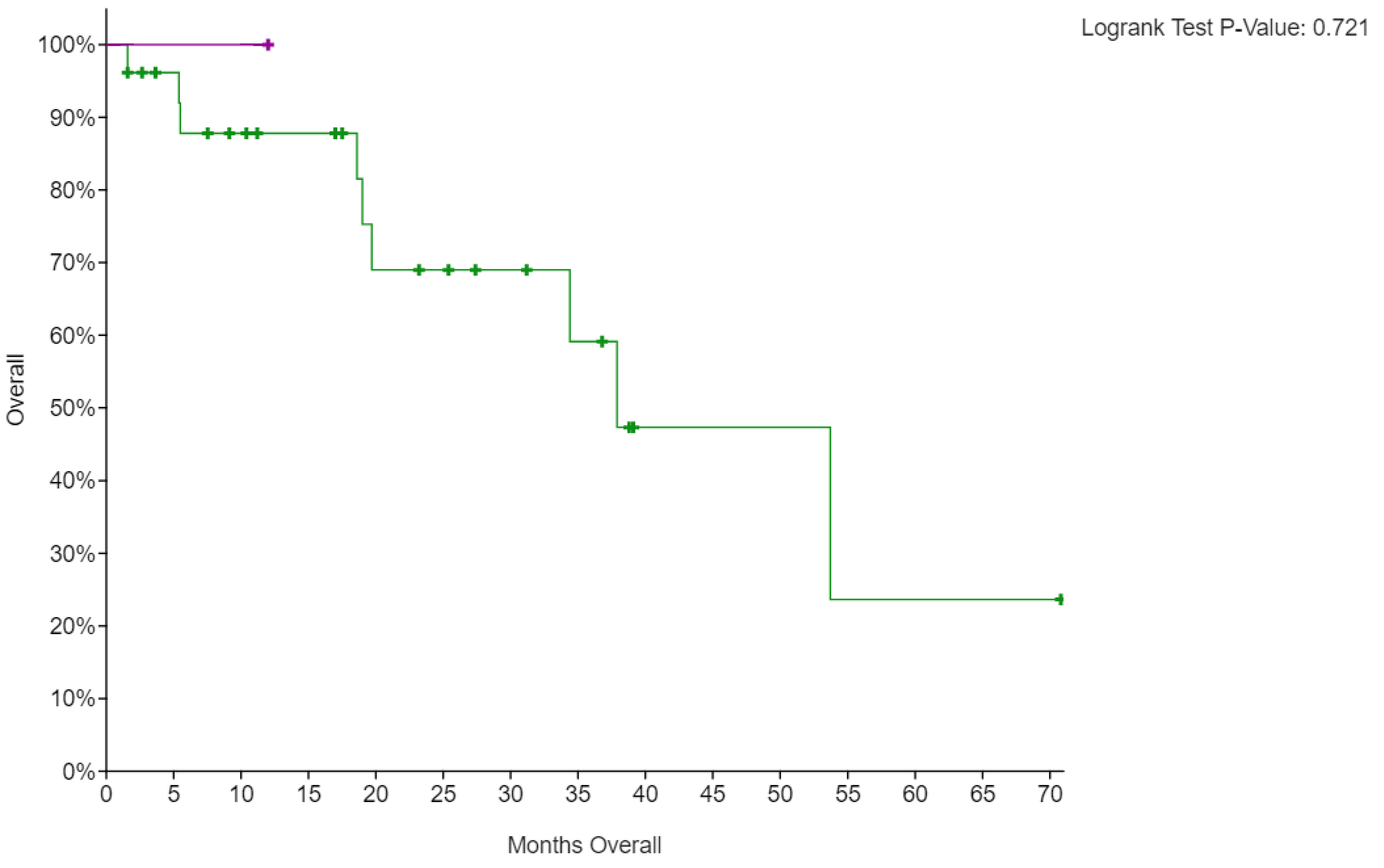

Overall

- FGFR3+ ATM-
- FGFR3+ ATM+

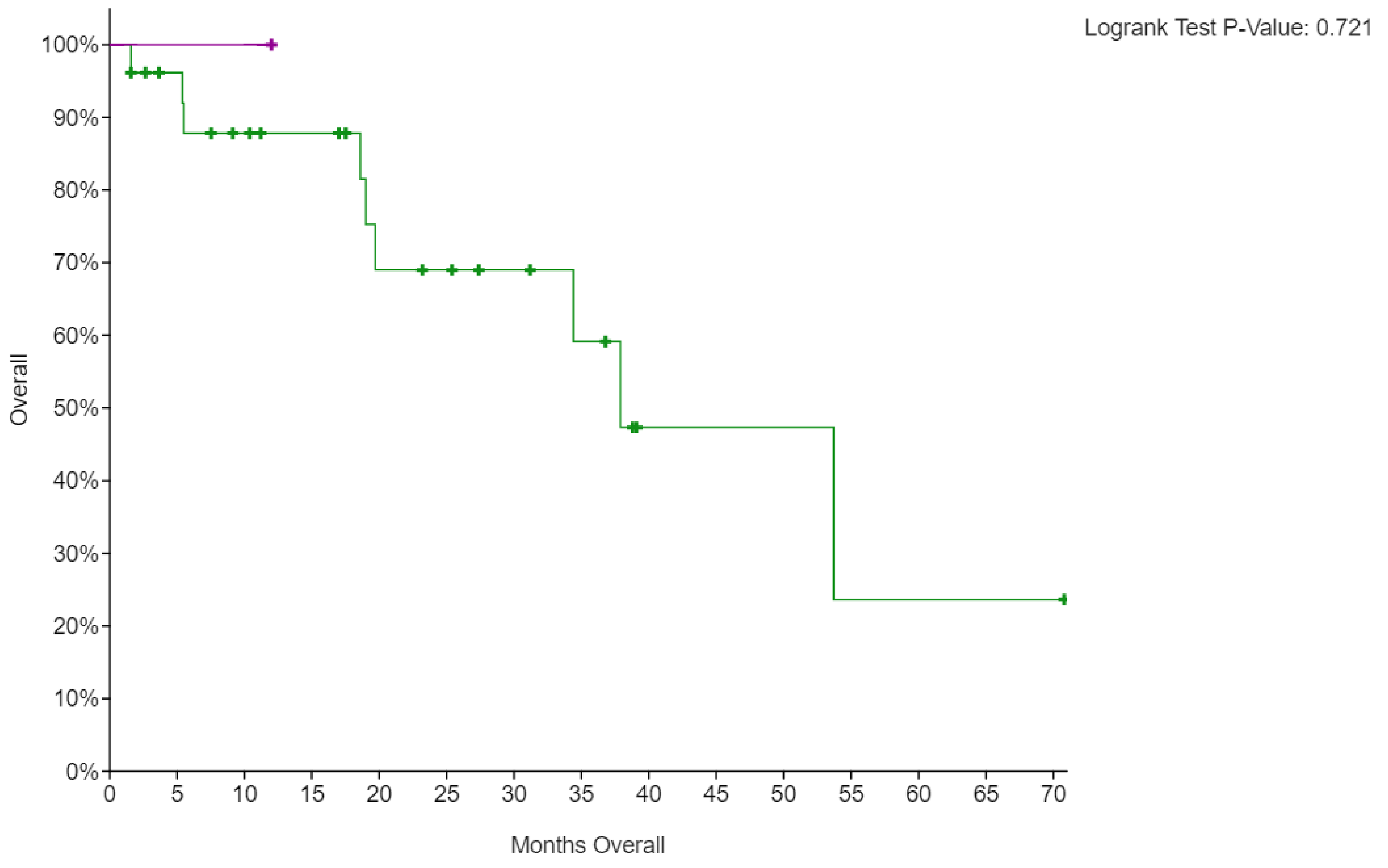

Overall

- FGFR3+ ATM-
- FGFR3+ ATM+

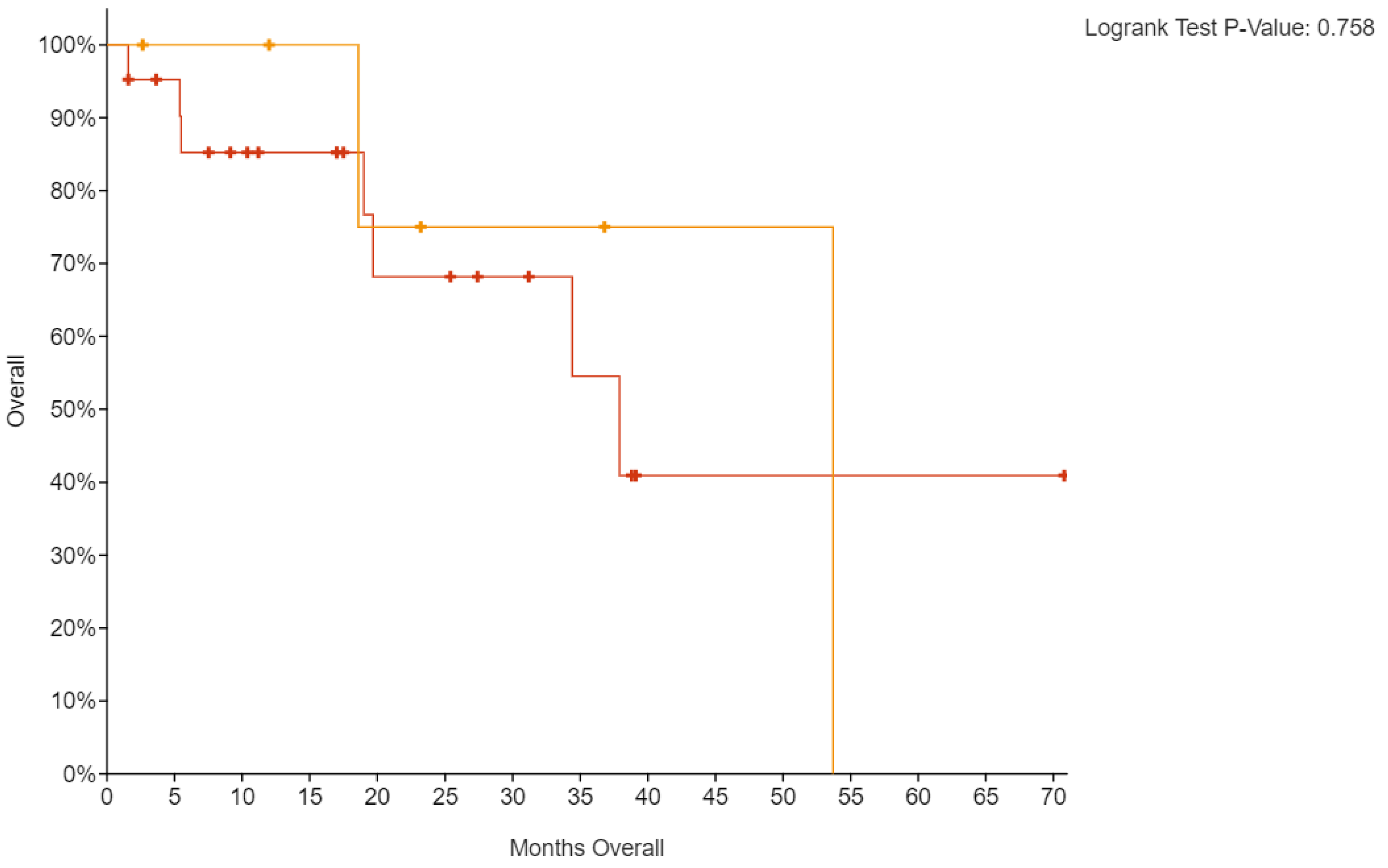

Overall

- FGFR3+ TP53-
- FGFR3+ TP53+

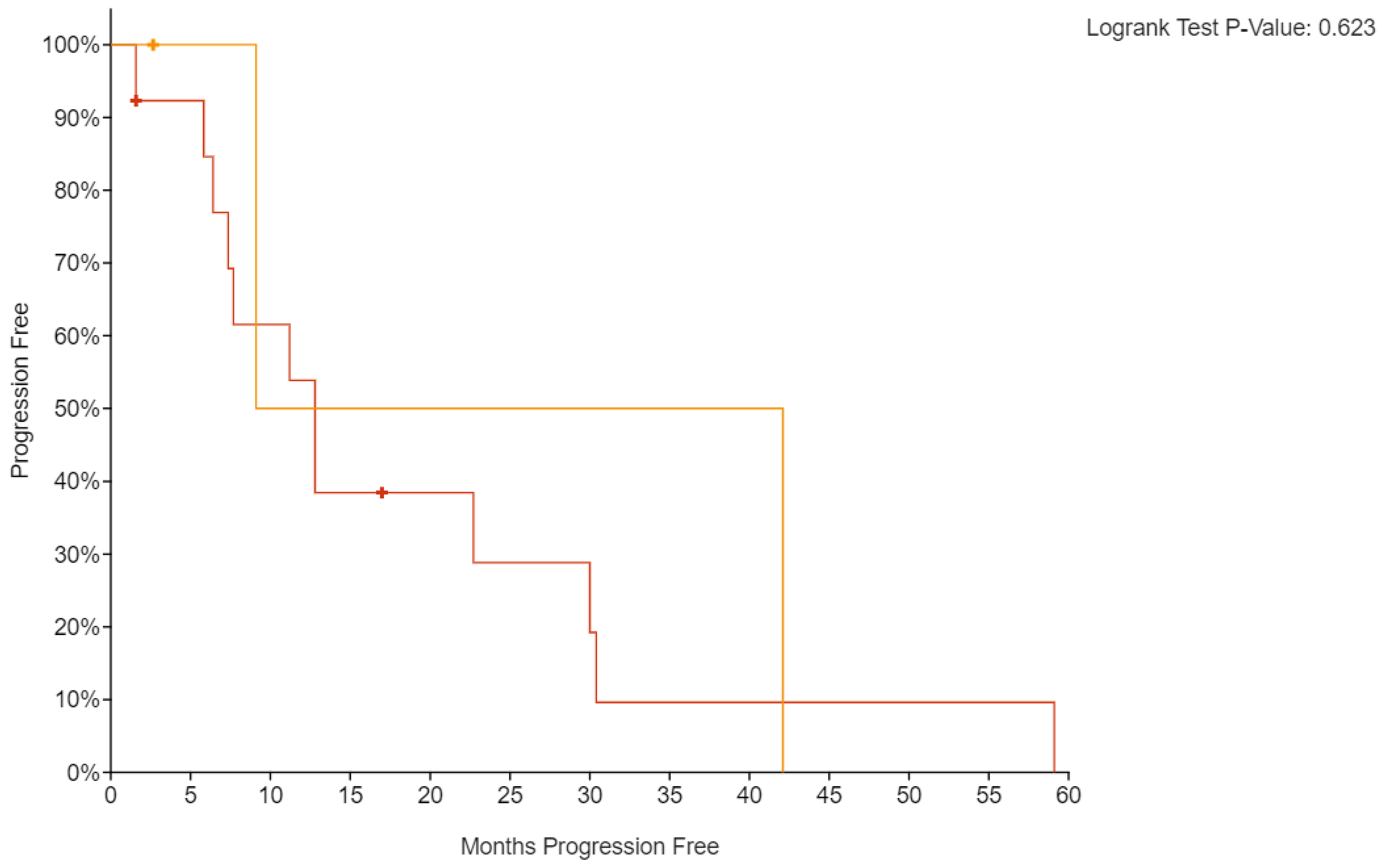

Progression Free

- FGFR3+ TP53-
- FGFR3+ TP53+

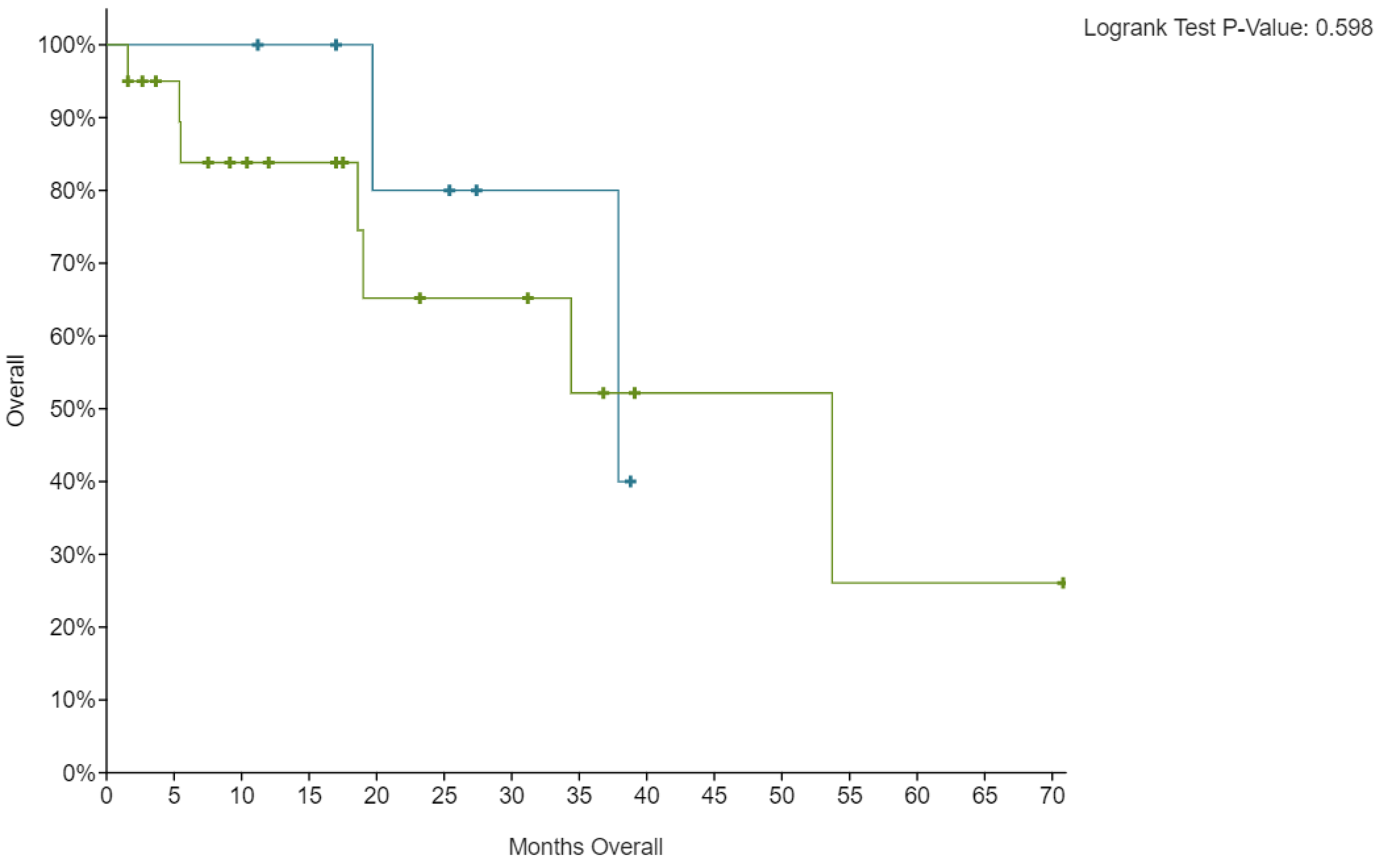

Overall

- FGFR3+ p14arf signaling-
- FGFR3+ p14arf signaling+

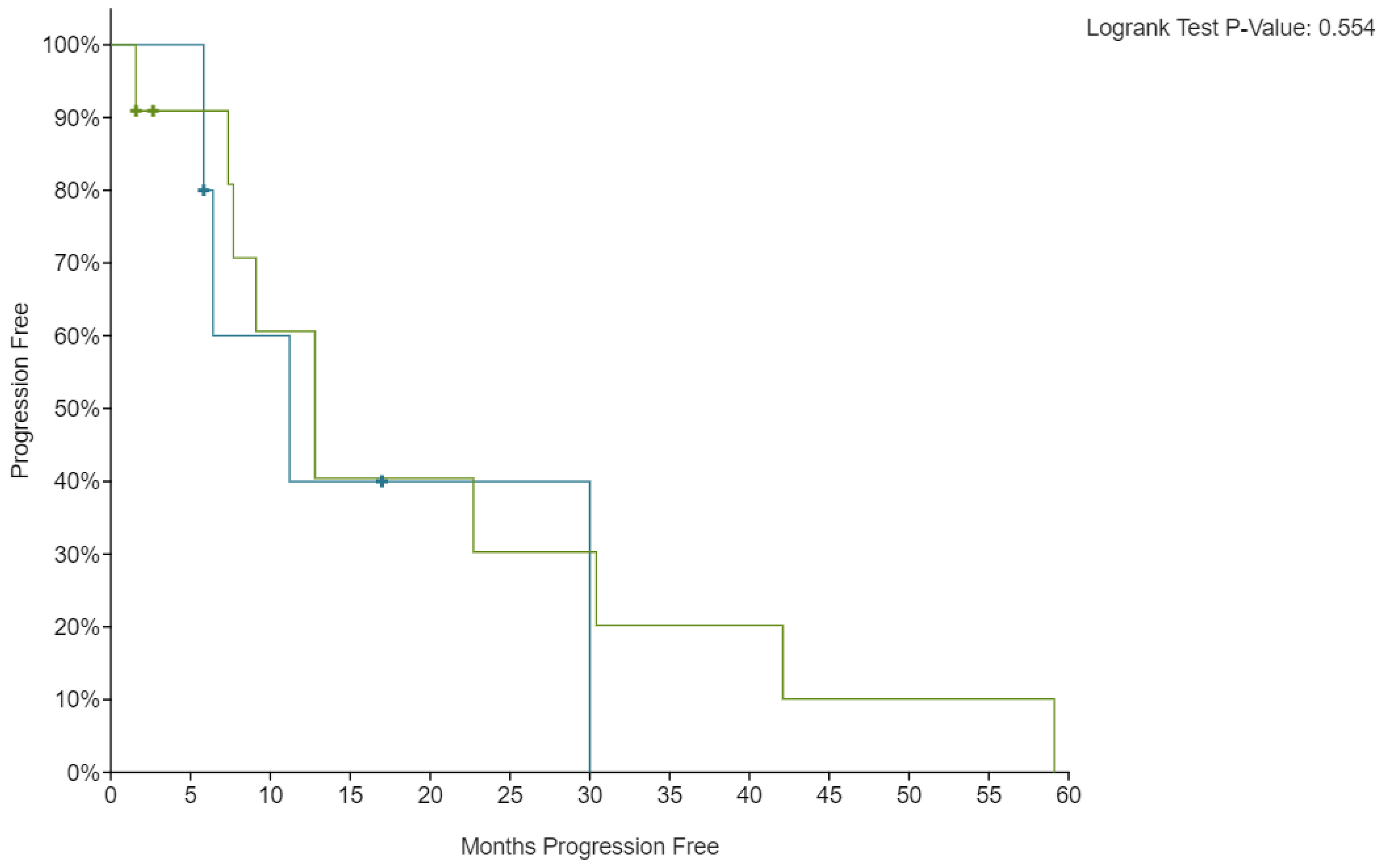

Progression Free

- FGFR3+ p14arf signaling-
- FGFR3+ p14arf signaling+

Supplementary Figure 4 – Telomere maintenance genes

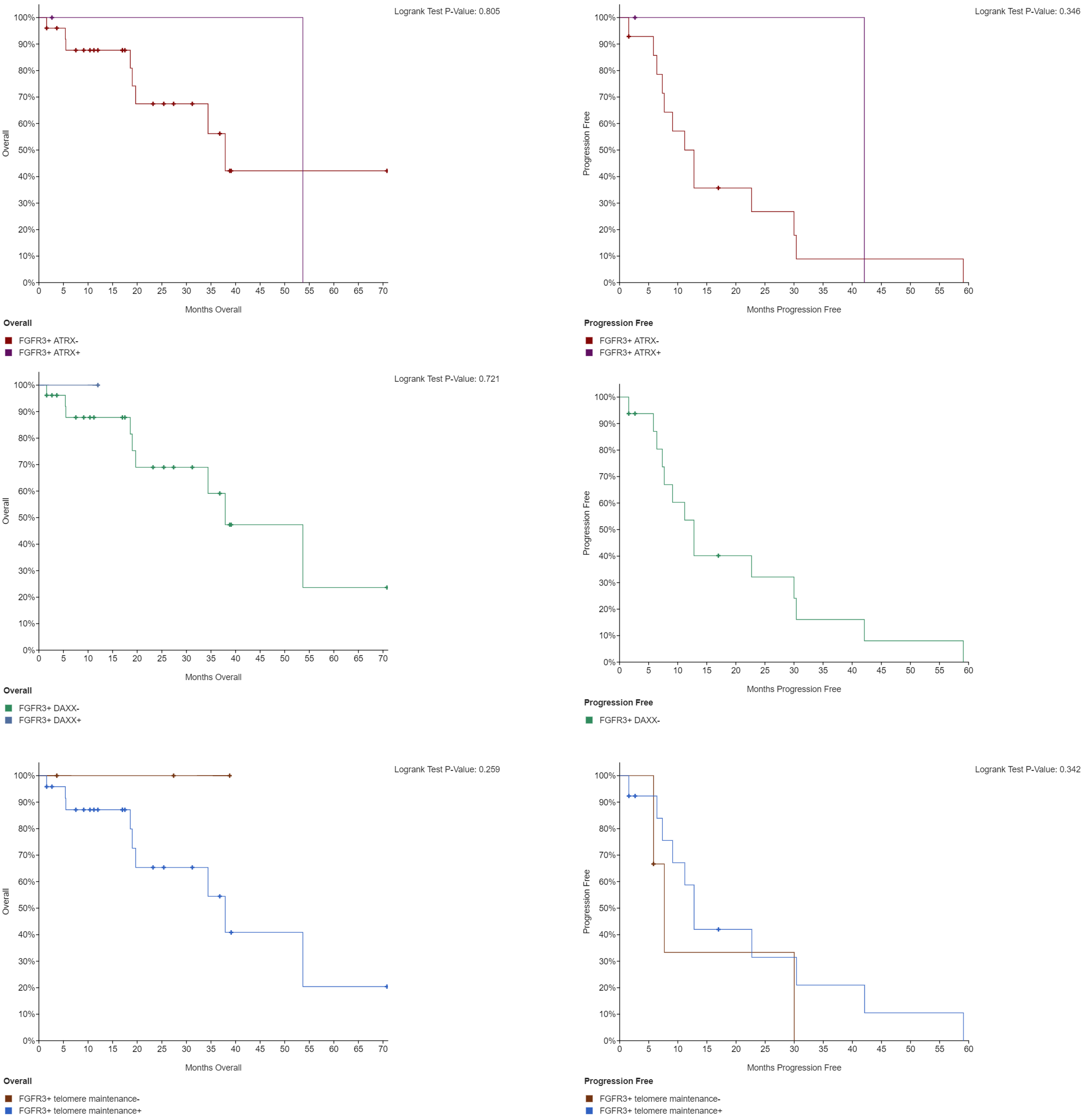

Supplement: Supplementary file 1 [file DataSheet_1.pdf]
